# RevCAR-mediated T-cell response against PD-L1-expressing cells turns suppression into activation

**DOI:** 10.1038/s41698-025-00828-6

**Published:** 2025-02-09

**Authors:** Eugenia Crespo, Liliana R. Loureiro, Antonia Stammberger, Lydia Hoffmann, Nicole Berndt, Anja Hoffmann, Claudia Dagostino, Karla E. G. Soto, Luise Rupp, Claudia Arndt, Martin Schneider, Claudia R. Ball, Michael Bachmann, Marc Schmitz, Anja Feldmann

**Affiliations:** 1 Institute of Radiopharmaceutical Cancer Research, Helmholtz-Zentrum Dresden-Rossendorf (HZDR), Dresden, Germany; 2 Institute of Immunology, Faculty of Medicine Carl Gustav Carus, TUD Dresden University of Technology, Dresden, Germany; 3 Department for Translational Medical Oncology, National Center for Tumor Diseases Dresden (NCT/UCC), a partnership between DKFZ, Faculty of Medicine and University Hospital Carl Gustav Carus, TUD Dresden University of Technology, and Helmholtz-Zentrum Dresden-Rossendorf (HZDR), Dresden, Germany; 4 Translational Medical Oncology, Faculty of Medicine and University Hospital Carl Gustav Carus, TUD Dresden University of Technology, Dresden, Germany; 5 Mildred Scheel Early Career Center, Faculty of Medicine Carl Gustav Carus, TUD Dresden University of Technology, Dresden, Germany; 6 Department of General, Visceral and Transplantation Surgery, Heidelberg University Hospital, Heidelberg, Germany; 7 Faculty of Biology, TUD Dresden University of Technology, Dresden, Germany; 8 German Cancer Consortium (DKTK), partner site Dresden, Dresden, Germany; 9 National Center for Tumor Diseases (NCT), partner site Dresden, Dresden, Germany

**Keywords:** Cancer models, Cancer immunotherapy

## Abstract

Applying CAR T-cell therapy to treat solid tumors is especially challenging due to the immunosuppressive tumor microenvironment (TME). While our modular RevCAR system enhances the safety and controllability of CAR T-cell therapy, effectively targeting solid tumors remains difficult. Since PD-L1 is an immune checkpoint frequently upregulated by cancer cells and their microenvironment, it is a relevant target for solid tumors. Here, we introduce a novel PD-L1 RevTM capable of redirecting RevCAR T-cells to specifically target and kill PD-L1-expressing tumor cells, becoming activated and secreting pro-inflammatory cytokines. This is shown in vitro with monolayer and 3D models, including patient-derived cultures, and in vivo. Furthermore, we demonstrate in vitro and in vivo an AND-gated targeting of cells simultaneously expressing PD-L1 and another tumor-associated antigen by the Dual RevCAR system. Our findings suggest that RevCAR-mediated targeting of PD-L1 could be a promising therapeutic approach for modulating the TME and improving solid tumor treatment.

## Introduction

Cancer is the second leading cause of death worldwide and remains one of the biggest challenges in healthcare^1^. Furthermore, as the longevity of humans increases, so does the prevalence of tumor diseases^2^. Even patients who survive cancer often have severe and long-lasting side effects from aggressive conventional treatments such as chemotherapy and radiation^3,4^. In recent years, immunotherapy has risen as another treatment avenue, harnessing and amplifying the power of the patient’s own immune system^5^. One of the most promising applications is autologous CAR T-cell therapy, in which the T-cells from the patient are genetically modified to express chimeric antigen receptors (CARs) and thus redirected to kill cells expressing tumor-associated antigens (TAAs) on their surface^6^. This kind of therapy has already shown remarkable results mainly in hematological malignancies, with six CAR products approved to treat cancer patients^7,8^. However, there are various risks associated with conventional CAR T-cell therapy, such as on-target-off-tumor effects and cytokine release syndrome^9,10^. These issues challenge the applicability of this therapy for a wide range of cancers, and safer approaches are needed^11,12^.

Conventional CAR T-cells, once inside the patient’s body, may continue to strike their target, even after the tumor cells have been eliminated. In contrast, the modular CAR systems developed by our group, UniCAR (Universal CAR) and RevCAR (Reverse UniCAR), require an adapter molecule named target module (TM or RevTM) for the killing to take place^13,14^. UniCAR or RevCAR T-cells alone are unable to bind directly to a TAA, meaning that in the case of adverse effects, the system can be effectively and rapidly switched off simply by stopping the infusion of the short-lived TM. The functionality and safety switch of the UniCAR system has already been demonstrated in an ongoing clinical trial for acute myeloid leukemia^15,16^. Both of our modular CAR systems are based on the interaction of a small peptide epitope (E5B9) derived from the human nuclear La/SS-B protein and a single chain fragment variable (scFv) that is able to bind to it^17^. In the RevCARs, the peptide epitope is on the extracellular domain, and the anti-La scFv is on the RevTM. Apart from the increased safety, adapter CARs may also circumvent some resistance mechanisms. Simply by adding a target module with a different antigen specificity, the same CAR T-cells can be used to target multiple antigens without reengineering (also known as OR-gated targeting), thereby eliminating tumor escape variants that have lost the original target. Given that TAAs are also expressed to a certain extent by healthy tissues, higher tumor specificity can be achieved by combinatorial targeting (also known as AND-gated targeting) using the Dual RevCAR system developed by us, in which the signaling and costimulatory domains of the RevCAR are divided into two different RevCAR constructs^14^. Dual RevCAR T-cells, therefore, require the presence of two different RevTMs simultaneously binding to two TAAs expressed on the surface of the target cells to become fully activated, sparing healthy tissues that express only one of the TAAs. The functionality of this AND-gated tumor targeting approach has been proven in vitro and in vivo, targeting different antigen pairs and tumor entities, such as prostate cancer, colorectal cancer, and glioblastoma^14,18–20^.

Despite the success of CAR T-cells in treating hematological malignancies, the landscape remains more challenging in the case of solid tumors, mainly because the immunosuppressive tumor microenvironment (TME) makes it very difficult for the T-cells to infiltrate, perform effectively, and destroy solid tumors^21–23^. Immune checkpoints such as PD-L1 and CTLA-4, which usually play an important role in regulating the immune system, are often upregulated by tumor cells and their environment as a way to hinder the immune response^24^. For this reason, immune checkpoint blocking antibodies are another form of immunotherapy often used, with nine approved products in the clinics, but the responses are heterogeneous, and resistance or adverse effects often develop^25–27^. There are several ongoing clinical trials combining CAR T-cells with PD-1/PD-L1 and CTLA-4 blockade^28^. Other interesting approaches, such as CAR T-cells that can secrete a PD‐L1-blocking scFv or a PD-L1-targeting chimeric switch receptor have been recently developed, the latter now going into clinical trials^29,30^. However, these strategies do not directly target PD-L1-expressing cells. Conversely, PD-L1 CARs have already been described, but these lack the additional safety that our modular system can offer^31^. Therefore, an approach that not only prevents immune cell suppression but that actively promotes the killing of immune-checkpoint-expressing cells in a safe and switchable manner would be beneficial.

For this purpose, we have developed a novel RevTM for the RevCAR system targeting the immune checkpoint PD-L1. In contrast to the antibody-based format that we commonly use, we have designed a PD-1 protein-derived molecule, taking advantage of the natural PD-1/PD-L1 interaction. We have assessed its functionality in several 2D and 3D in vitro models, as well as in vivo. We have also tested a combinatorial AND-gated tumor targeting approach with the TAA PSCA and PD-L1, predicting a versatile and precise application of PD-L1 targeting by the RevCAR system in a wide range of solid tumor entities.

## Results

### Design and production of the PD-L1 RevTM

**Fig. 1:**
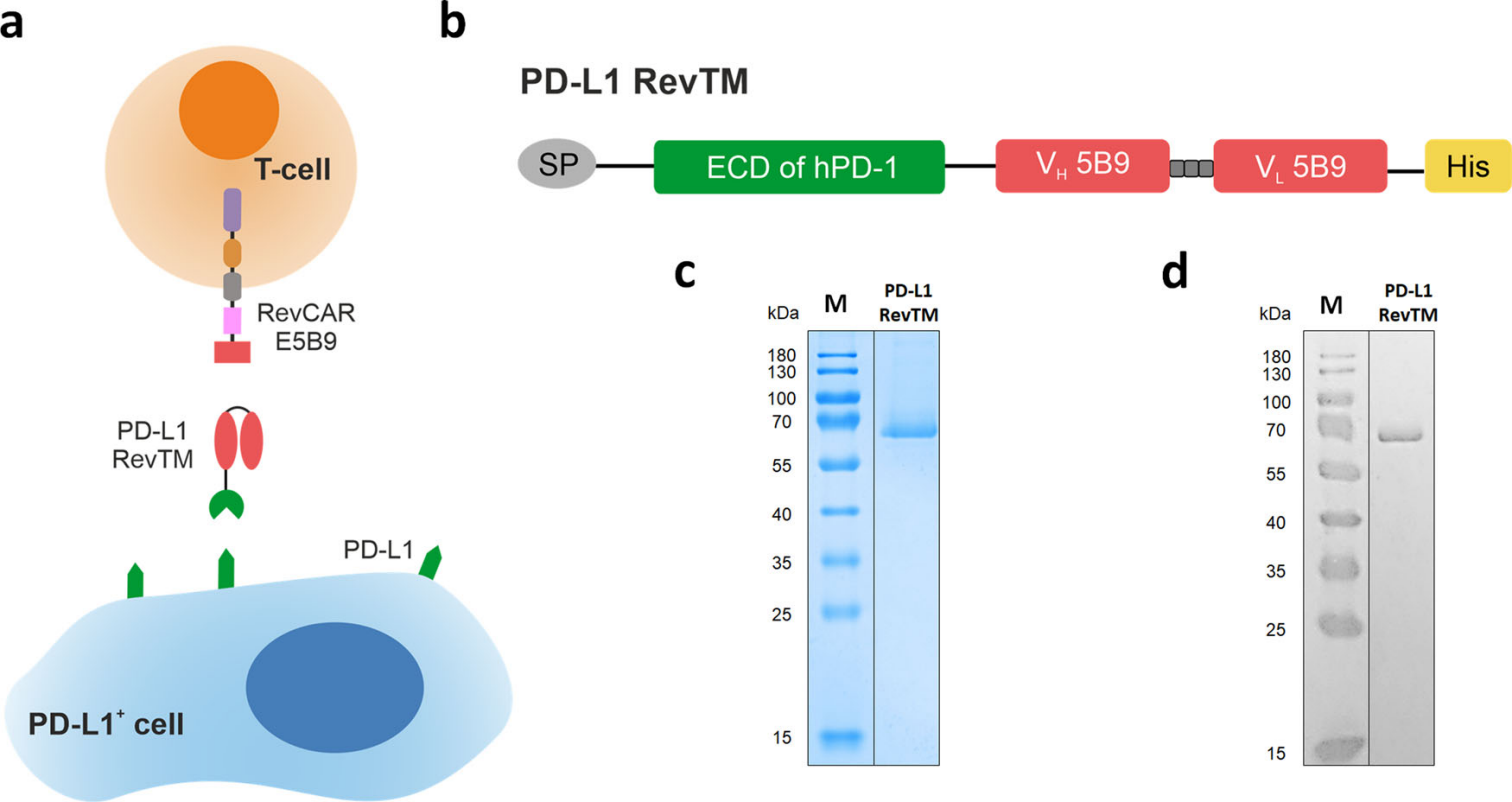
Design and purification of PD-L1 RevTM. **a** For redirection of RevCAR T-cells towards PD-L1-expressing cells, the novel PD-L1 RevTM was generated. PD-L1 RevTM is depicted in red and green. RevCAR E5B9 contains the intracellular CD3z signaling domain (purple) and CD28 co-stimulatory domain (brown), a transmembrane domain (gray), hinge domain (pink), and the peptide epitope E5B9 (red). **b** Structure of the PD-L1 RevTM, containing the extracellular domain (ECD) of human PD-1 (green), which naturally binds to its ligand PD-L1, connected through a linker to an anti-La (5B9) scFv (red). The RevTM also includes a signal peptide (SP, gray) for its secretion and a 6x histidine tag (His, yellow) for purification and detection. PD-L1 RevTM was expressed by stably transduced 3T3 cells and purified from the cell culture supernatant via Ni-NTA chromatography. The eluted protein was separated by SDS-PAGE and visualized by **c** Coomassie staining and **d** Western blotting using an anti-His antibody. M = molecular weight marker.

A novel protein-based RevTM, termed PD-L1 RevTM, was developed to target PD-L1 with the RevCAR system (Fig. 1a). RevCAR-E5B9-28/3z T-cells, which from now on will be referred to as RevCAR T-cells, contain in their extracellular domain the small peptide epitope E5B9 of the human nuclear La/SS-B protein^13^. Therefore, the RevTM contains the anti-La (5B9) scFv that binds to this peptide epitope (Fig. 1b). For binding to PD-L1, on the other hand, the PD-L1 RevTM contains the extracellular domain (ECD) of the human PD-1 protein, taking advantage of the natural interaction between the PD-1 receptor and its ligand. The PD-1 ECD and anti-La (5B9) scFv are connected via a short peptide linker. The resulting RevTM was cloned into a lentiviral vector which was used to stably transduce the murine embryonic fibroblast cell line 3T3 for permanent RevTM expression. Due to the N-terminal signal peptide (SP) the RevTM was secreted to the culture medium, which was harvested for purification of the PD-L1 RevTM by Ni-NTA affinity chromatography using the C-terminal His tag. The purified product was analyzed by SDS-PAGE followed by Coomassie staining (Fig. 1c) or Western blot (Fig. 1d). Both the Coomassie staining and the anti-His Western blot showed a single band at ~60 kDa, confirming a successful purification of the PD-L1 RevTM. Although the theoretical size of the protein is ~45 kDa, the higher molecular weight may be due to aberrant running behavior and post-translational modifications, most likely glycosylation. After the production and biochemical characterization of the PD-L1 RevTM, the affinity and functionality could be assessed.

### Binding assessment and specific killing of PD-L1-expressing tumor cells in monolayer

**Fig. 2:**
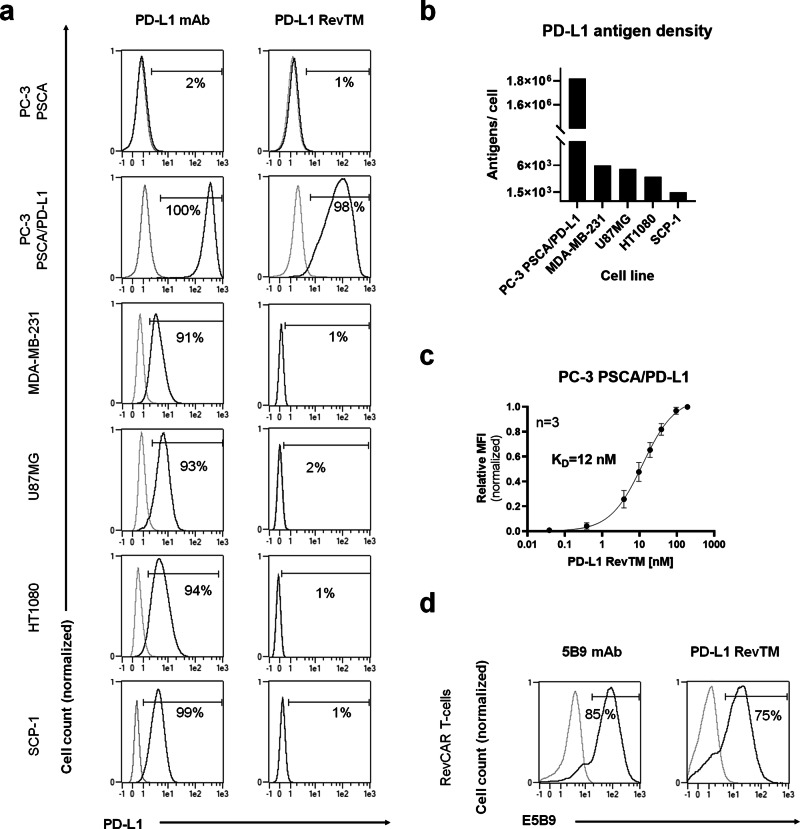
PD-L1 expression on target cells and binding of PD-L1 RevTM to target cells and RevCAR T-cells. **a** PC-3 PSCA, PC-3 PSCA/PD-L1, MDA-MB-231, U87MG, HT1080, and SCP-1 cells were stained with anti-PD-L1 monoclonal antibody (mAb) or PD-L1 RevTM and detected via goat anti-mouse Pacific Blue mAb and anti-His-PE mAb as the secondary, respectively, using flow cytometry. **b** PD-L1 density on the surface of target cells was quantified after staining them with the anti-PD-L1 mAb, using the flow cytometry-based QIFIKIT. **c** Titration of PD-L1 RevTM binding to PC-3 PSCA/PD-L1 cells was performed, and *K*_D_ values were determined. Data are plotted as normalized MFI ± SD for three individual experiments. **d** The expression of RevCAR E5B9 on modified T-cells was confirmed by staining RevCAR T-cells with the anti-La (5B9) mAb as positive control. Subsequently, RevCAR T-cells were stained with PD-L1 RevTM to show its binding capability to E5B9-expressing RevCAR T-cells. **a**, **d** Stained cells (black curves) and corresponding controls (gray curves) are displayed as histograms, and the percentage of positively stained cells is indicated. Results for one representative binding assay are shown.

To find suitable models for PD-L1 targeting, several cell lines were tested for PD-L1 expression by a flow cytometry-based binding assay with an anti-PD-L1 monoclonal antibody (Fig. 2a). The human breast cancer cell line MDA-MB-231, fibrosarcoma cell line HT1080, glioblastoma cell line U87MG and immortalized mesenchymal cell line SCP-1 were positive for PD-L1 expression, while the prostate cancer cell line PC-3, which had already been stably transduced to express human PSCA, was PD-L1 negative. In order to generate a stable and high PD-L1-expressing cell line, PC-3 PSCA cells were transduced to overexpress PD-L1, originating a new cell line named PC-3 PSCA/PD-L1. The ability of the PD-L1 RevTM to bind to PD-L1-expressing cell lines was also assessed by flow cytometry.

As expected, the PD-L1 RevTM did not bind to PC-3 PSCA cells lacking PD-L1 expression, but a binding could be detected on PC-3 PSCA/PD-L1 cells, proving the specificity of the RevTM for PD-L1. However, for PD-L1-positive cell lines MDA-MB-231, U87MG, HT1080 and SCP-1, a binding could not be detected for the PD-L1 RevTM with this method, probably due to the low PD-L1 density on these cells (Fig. 2b). We found that PC-3 PSCA cells overexpressing PD-L1 had 1.8 × 10^6^ antigens per cell, compared to 1.5 × 10^3^ to 6 × 10^3^ in the case of cell lines naturally expressing PD-L1. Using PC-3 PSCA/PD-L1 cells, a titration was done with different concentrations of PD-L1 RevTM and a *K*_D_ of 12 nM was determined (Fig. 2c). On the other side, binding of the PD-L1 RevTM to RevCAR T-cells was also demonstrated by flow cytometry, while expression of RevCARs E5B9 on genetically modified T-cells was confirmed by their detection with the anti-La (5B9) antibody (Fig. 2d).

**Fig. 3:**
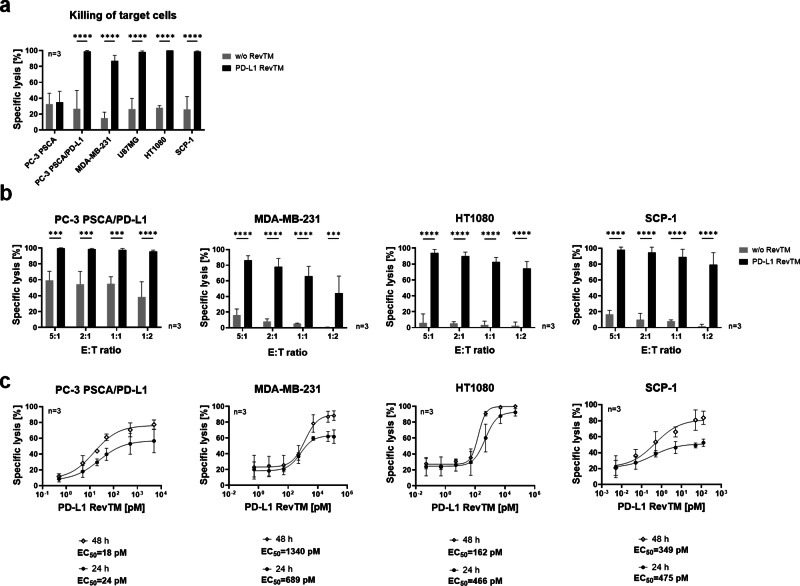
Cytotoxic functionality in vitro of RevCAR system redirected against PD-L1-expressing monolayer target cells. **a** Killing assay of PD-L1-negative (PC-3 PSCA), PD-L1-overexpressing (PC-3 PSCA/PD-L1) and endogenously PD-L1-expressing (MDA-MB-231, U87MG, HT1080 and SCP-1) cell lines with the RevCAR system. Target cells (5 × 10^3^) were co-cultured for 48 h with RevCAR T-cells (2.5 × 10^4^) at the effector to target cell ratio (E:T) of 5:1 in the presence or absence of PD-L1 RevTM, and specific lysis was determined by luciferase-based cytotoxicity assay. **b** Target cells and RevCAR T-cells were co-cultured for 48 h at different E:T ratios in the presence or absence of PD-L1 RevTM and specific lysis was determined by luciferase-based cytotoxicity assay. **a**, **b** Data for three individual T-cell donors are shown as mean ± SD. Statistical significance was assessed by two-way ANOVA followed by Šídák’s multiple comparisons test; *P* ≤ 0.001 (***), *P* ≤ 0.0001 (****). **c** Target cells (5 × 10^3^) were incubated with RevCAR T-cells (E:T = 5:1) and different concentrations of PD-L1 RevTM for 24 h and 48 h and specific lysis was determined. Dose-response curves were plotted as mean specific lysis ± SD from three individual T-cell donors and half maximal effective concentration values (EC_50_) were determined.

To determine if the PD-L1 RevTM can redirect RevCAR T-cells to kill PD-L1-expressing target cells, target cells were co-cultured with RevCAR T-cells in the presence or absence of PD-L1 RevTM for 48 h as shown in Fig. 3a. All five PD-L1-expressing target cells tested can be effectively and significantly killed by RevCAR T-cells in the presence of the novel PD-L1 RevTM.

In contrast, as predicted, no specific target cell lysis was observed in the absence of the PD-L1 RevTM. Furthermore, the PD-L1-negative cell line PC-3 PSCA could not be killed either with or without PD-L1 RevTM (Fig. 3a). To further assess the efficiency of the system, different effector to target cell (E:T) ratios were used (Fig. 3b). The redirected RevCAR T-cells were able to successfully and significantly kill the target cells expressing different PD-L1 density levels even at a low E:T ratio of 1:2. Different amounts of PD-L1 RevTM were used to calculate the half-maximum effective concentration (EC_50_) (Fig. 3c), revealing the EC_50_ of PD-L1 RevTM for killing of PC-3 PSCA/PD-L1 to be in the low pM range, and for the target cells with a moderate PD-L1 expression, EC_50_ values were in the high pM to low nM range. While EC_50_ values stayed similar at different time points, an increase in maximum killing was observed at 48 h compared to 24 h. Altogether, the results so far indicate that PD-L1 RevTM can bind to PD-L1-expressing monolayer target cells of different entities and redirect RevCAR T-cells to specifically kill them with high efficiency in a RevTM- and target-dependent manner.

### RevCAR T-cell activation, differentiation, and cytokine release

**Fig. 4:**
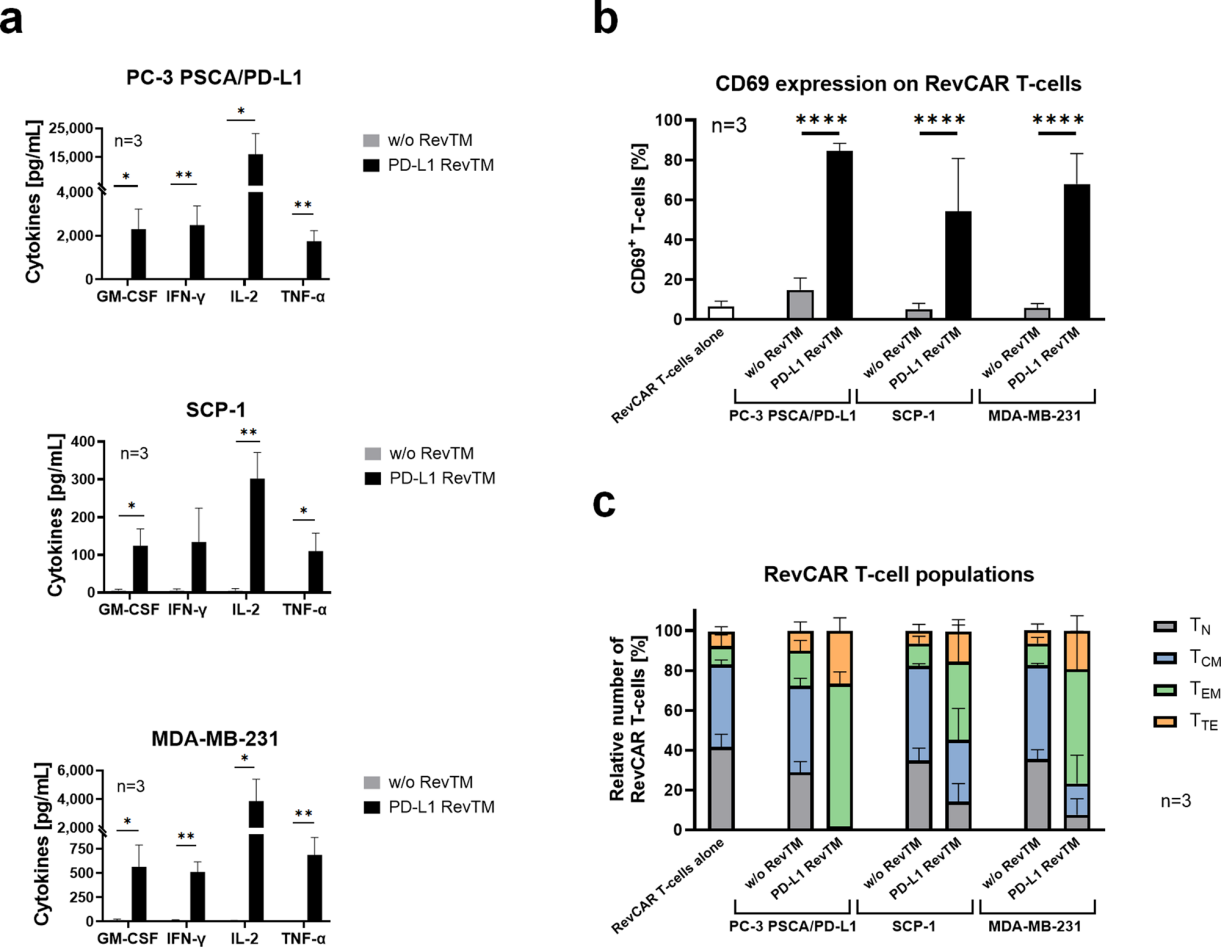
Characterization of RevCAR T-cells redirected against PD-L1-expressing monolayer target cells with PD-L1 RevTM. **a** Target cells (5 × 10^3^) were co-cultured with RevCAR T-cells (E:T = 5:1) in the presence or absence of PD-L1 RevTM for 24 h. The supernatant was harvested, and the secretion of pro-inflammatory cytokines was determined. Statistical significance was assessed by Student’s independent *t* test. *P* ≤ 0.05 (*), *P* ≤ 0.01 (**). **b** RevCAR T-cells were seeded in the absence of target cells, in the presence of target cells alone, or in the presence of target cells and PD-L1 RevTM for 24 h. Then, the RevCAR T-cells were stained with CD69 antibody. Statistical significance was assessed by two-way ANOVA followed by Tukey’s multiple comparisons test; *P* ≤ 0.0001 (****). **c** Co-cultures were set as described in *b*, but in this case, after incubation, the T-cells were stained with CD62L and CD45RO antibodies, and according to the expression levels of these markers, RevCAR T-cells were divided into four groups of increasing differentiation: Naive (T_N_): CD62L^high^ and CD45RO^low^; Central memory (T_CM_): high expression of both markers. Effector memory (T_EM_): CD62L^low^ and CD45RO^high^; and Terminal effector (T_TE_): low expression of both markers. **a**–**c** Data for three individual T-cell donors are shown as mean ± SD.

Once it was successfully established that the PD-L1 RevTM can redirect RevCAR T-cells to kill PD-L1-expressing cells in monolayer, we wanted to gain more information about the phenotype of the RevCAR T-cells and their capacity to release pro-inflammatory cytokines. For this, we set up co-cultures of the target cells with RevCAR T-cells in the presence or absence of PD-L1 RevTM. The supernatant was harvested after 24 h and cytokine concentration was determined. RevCAR T-cells significantly released the cytokines GM-CSF, IFN-γ, TNF-α, and IL-2 upon PD-L1 recognition on target cells via the PD-L1 RevTM as shown in Fig. 4a. For PC-3 PSCA/PD-L1, the cytokine secretion was stronger than for the MDA-MB-231 and the SCP-1 cells, which correlates with the PD-L1 density (Fig. 2b). To determine the activation and phenotype status of RevCAR T-cells upon their redirection towards PD-L1-expressing target cells via the PD-L1 RevTM, a co-culture was once again established and the RevCAR T-cells were harvested and stained after 24 h. It could be observed that CD69 expression on RevCAR T-cells was only significantly increased in the presence of PD-L1 RevTM for all three PD-L1-expressing cell lines (Fig. 4b), confirming the specific activation of RevCAR T-cells upon PD-L1 recognition on target cells.

Along these lines, a more detailed phenotypical analysis of the RevCAR T-cell subpopulations for all tested PD-L1 target cell lines revealed that the portion of naive or central memory T-cells decreased in the presence of PD-L1 RevTM compared to the control without RevTM, while the portion of effector memory and terminal effector T-cells considerably increased (Fig. 4c). Apparently, a shift from naive and central memory T-cells towards effector T-cell subtypes takes place upon redirection of RevCAR T-cells against PD-L1 target cells via PD-L1 RevTM. These data, along with the killing results previously obtained, confirm that RevCAR T-cells, in the presence of PD-L1-positive target cells and PD-L1 RevTM, are specifically activated and release relevant pro-inflammatory cytokines, which are essential to obtain an effective and controlled attack against PD-L1-positive tumors.

### Specific infiltration and redirection of RevCAR T-cells in PD-L1-expressing spheroids

**Fig. 5:**
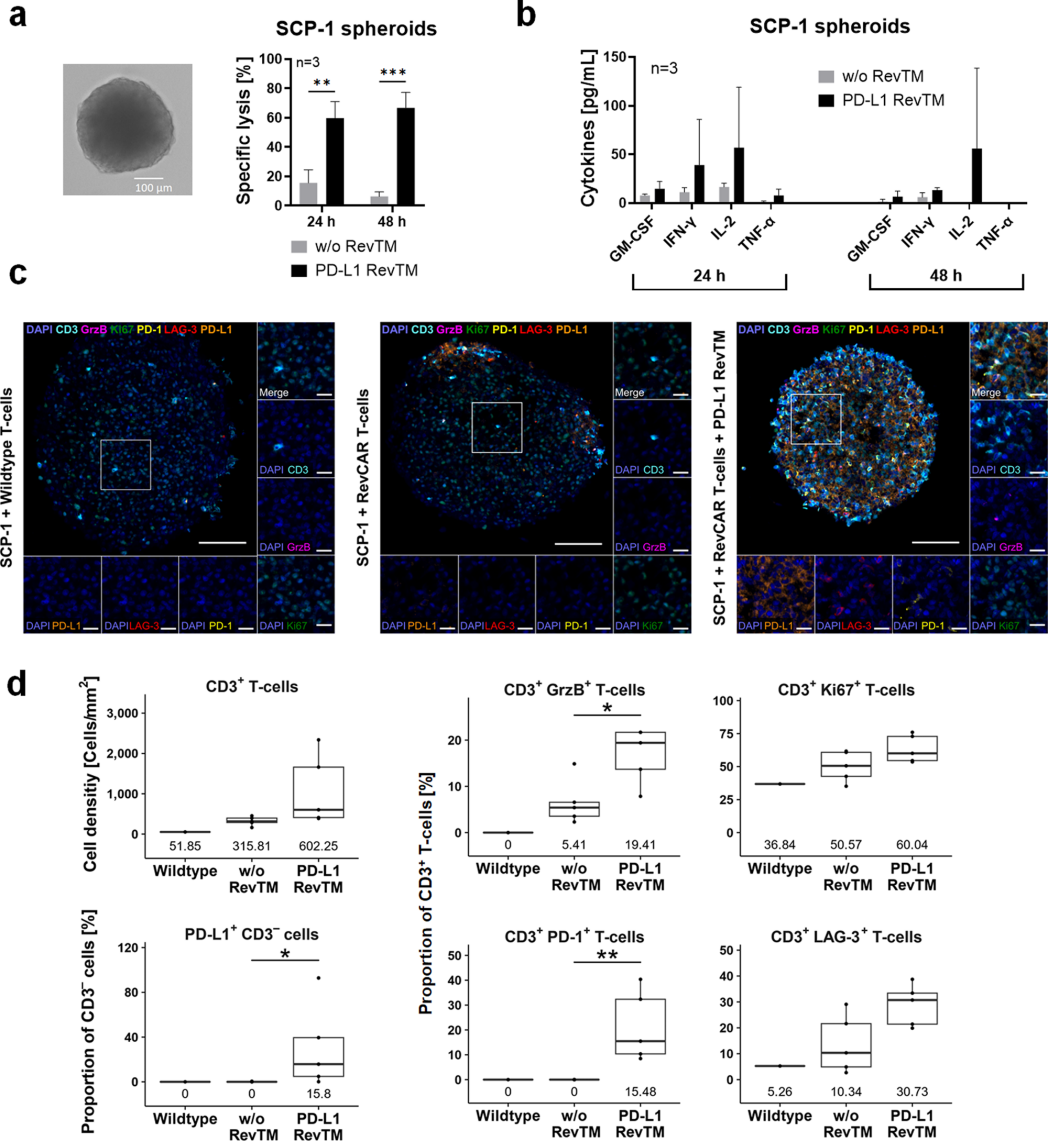
Killing and infiltration of SCP-1 spheroids and cytokine release by RevCAR T-cells in the presence of PD-L1 RevTM. **a** SCP-1 spheroids were established from 5 × 10^3^ seeded cells for 48 h (as shown in the brightfield microscope image). Afterwards, RevCAR T-cells were added (E:T = 5:1) in the presence or absence of PD-L1 RevTM, and after 24 h or 48 h co-culture, specific lysis was determined by luciferase-based cytotoxicity assays. Data for three individual T-cell donors are shown as mean ± SD. Statistical significance was assessed by Student’s independent *t* test. *P* ≤ 0.01 (**), *P* ≤ 0.001 (***). **b** After a 24 or 48 h co-culture of SCP-1 spheroids (5 × 10^3^ cells) with RevCAR T-cells (E:T = 5:1) in the presence or absence of PD-L1 RevTM, the supernatant was collected and cytokine secretion was evaluated. Data for three individual T-cell donors are shown as mean ± SD. **c** SCP-1 spheroids (6 × 10^4^ cells) were established for 48 h and afterwards co-cultured with either untransduced T-cells, RevCAR T-cells alone or RevCAR T-cells + PD-L1 RevTM. After 24 h, the spheroids were stained for CD3, Ki67, GrzB, PD-1, LAG-3, PD-L1, and DAPI. The scale bars in the overview image and the magnifications indicate 100 µm and 20 µm, respectively. **d** The cell density [Cells/mm^2^] of CD3^+^ T-cells and proportions [%] of CD3^+^ T-cells and PD-L1^+^ CD3^-^ cells and their medians are shown (Wildtype: *n* = 1; RevCAR T-cells with or without RevTM: *n* = 5). Statistical differences were identified using the unpaired Wilcoxon test; *P* ≤ 0.05 (*), *P* ≤ 0.01 (**).

To test if the RevCAR T-cells redirected by PD-L1 RevTM are able to infiltrate, disintegrate, and kill spheroids, we used a 3D model of the SCP-1 cell line as proof of concept. After forming stable SCP-1 spheroids (Fig. 5a), RevCAR T-cells were added in the presence or absence of PD-L1 RevTM and, after a 24 h or 48 h co-culture, specific lysis and cytokine release were determined (Fig. 5a, b). It was observed that PD-L1 RevTM induced the killing of the spheroids, and the redirected RevCAR T-cells specifically released pro-inflammatory cytokines (Fig. 5b). No significant difference was observed between the 24 h and 48 h incubations. Next, we wanted to further assess what was happening inside the spheroids. For this, once again, we carried out spheroid formation and afterward added either untransduced (Wildtype) T-cells, RevCAR T-cells alone, or RevCAR T-cells and PD-L1 RevTM. After the co-culture, a multiplex immunohistochemistry staining was performed and quantitatively analyzed to determine whether T-cells are present and get activated inside the spheroid (Fig. 5c, d). RevCAR T-cells were tracked via the T-cell marker CD3, showing that, indeed, a considerable infiltration in the PD-L1 spheroids was only observed in the presence of the PD-L1 RevTM. In addition, we stained PD-1 and LAG-3 to analyze whether the infiltrated T-cells got activated, granzyme B (GrzB) for analysis of T-cell cytotoxicity, and Ki67 as a proliferation marker. Our data indicates that the redirected RevCAR T-cells not only infiltrated the spheroids but also got significantly activated, proliferated, and induced cancer cell lysis mediated by GrzB. Furthermore, PD-L1 expression on SCP-1 cells increased upon cross-linkage to RevCAR T-cells in the presence of PD-L1 RevTM (CD3 negative cells, Fig. 5d). Altogether, these results show that PD-L1 targeting is possible not only in a 2D but also in a 3D tumor model, confirming a specific infiltration and activation of RevCAR T-cells in the presence of PD-L1 RevTM.

### Killing of patient-derived tumor spheroids by the novel PD-L1-specific RevCAR system

**Fig. 6:**
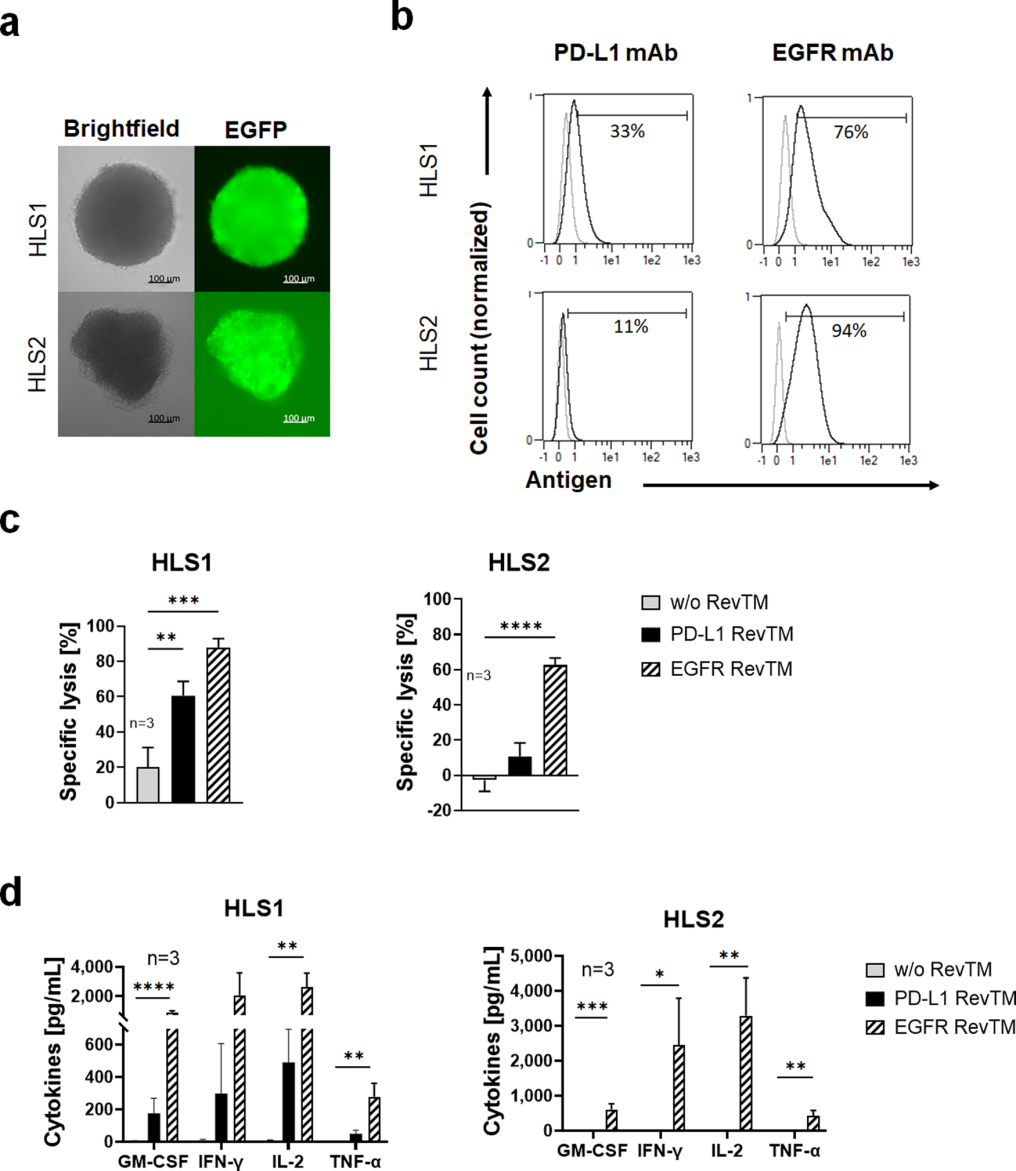
Specific killing of PD-L1- and EGFR-positive patient-derived sarcoma spheroids by RevCAR T-cells in the presence of PD-L1 RevTM or EGFR RevTM. **a** Microscopy images of patient-derived human liposarcoma spheroid cultures HLS1 and HLS2, which have been transduced to express luciferase, with EGFP as a reporter gene. **b** HLS1 and HLS2 spheroids were dissociated, stained with PD-L1 or EGFR mAb (as control), and analyzed with flow cytometry. **c** HLS1 and HLS2 spheroids (5 × 10^3^ cells) were co-cultured with RevCAR T-cells (E:T = 5:1) in the presence or absence of PD-L1 RevTM or EGFR RevTM (as control) for 24 h, after which specific lysis was assessed by luciferase-based cytotoxicity assays. Data for three individual T-cell donors are shown as mean ± SD. Statistical significance was assessed by one-way ANOVA followed by Tukey’s multiple comparisons test; *P* ≤ 0.01 (**), *P* ≤ 0.001 (***), *P* ≤ 0.0001 (****). **d** HLS1 and HLS2 spheroids were co-cultured with RevCAR T-cells in the presence or absence of PD-L1 RevTM or EGFR RevTM for 24 h, after which the supernatant was harvested, and cytokine secretion was detected. Data for three individual T-cell donors are shown as mean ± SD. Statistical significance was assessed by one-way ANOVA with Šídák’s multiple comparison test; *P* ≤ 0.05 (*), *P* ≤ 0.01 (**), *P* ≤ 0.001 (***), *P* ≤ 0.0001 (****).

To further assess the clinical relevance of the RevCAR system targeting PD-L1, we wanted to test our approach not only in established cell lines but also with patient-derived models. Therefore, we used expandable spheroids derived from liposarcoma tumors of two patients, which we termed HLS1 and HLS2. Patient-derived spheroid cultures can retain some of the properties of the original tumor, i.e., histological properties and functional heterogeneity, which are often lost in long-established monolayer cell lines^32^. Firstly, the spheroids were transduced with luciferase in order to use them as targets in luciferase-based cytotoxicity assays. Images of the spheroids are shown in brightfield and the EGFP channel, since the latter was used as a reporter gene for luciferase expression (Fig. 6a). Next, as assessed by flow cytometry, PD-L1 was detected on 33% of HLS1 cells but on only 11% of HLS2 cells of the patient-derived spheroid cultures (Fig. 6b). In addition, as a control, we assessed the expression of epidermal growth factor receptor (EGFR), which is often upregulated in liposarcoma, and for which we have already described a RevTM^19^. We found that both cultures expressed EGFR, concretely 76% of the HLS1 cells and 94% of the HLS2 cells (Fig. 6b). Remarkably, assessing the killing potential of PD-L1 and EGFR targeting with the RevCAR system we found that HLS1 can be significantly killed with either PD-L1 RevTM or EGFR RevTM. The percentage of killing was higher in the case of EGFR targeting, which is in line with the antigen expression (Fig. 6c). In the case of HLS2, we only observed a significant lysis for EGFR targeting, which is also expected since the PD-L1 expression by this culture was very low. These findings were further supported by an assessment of cytokine secretion profiles. We discovered that EGFR RevTM triggered a significant secretion of pro-inflammatory cytokines by the RevCAR T-cells in the presence of either HLS1 or HLS2. PD-L1 RevTM, on the other hand, induced a lower cytokine secretion in co-culture with HLS1 (Fig. 6d), but none with HLS2 in accordance with the low or marginal PD-L1 expression profile on these patient-derived spheroids.

### AND-gated targeting of PD-L1 and PSCA using Dual RevCAR T-cells

**Fig. 7:**
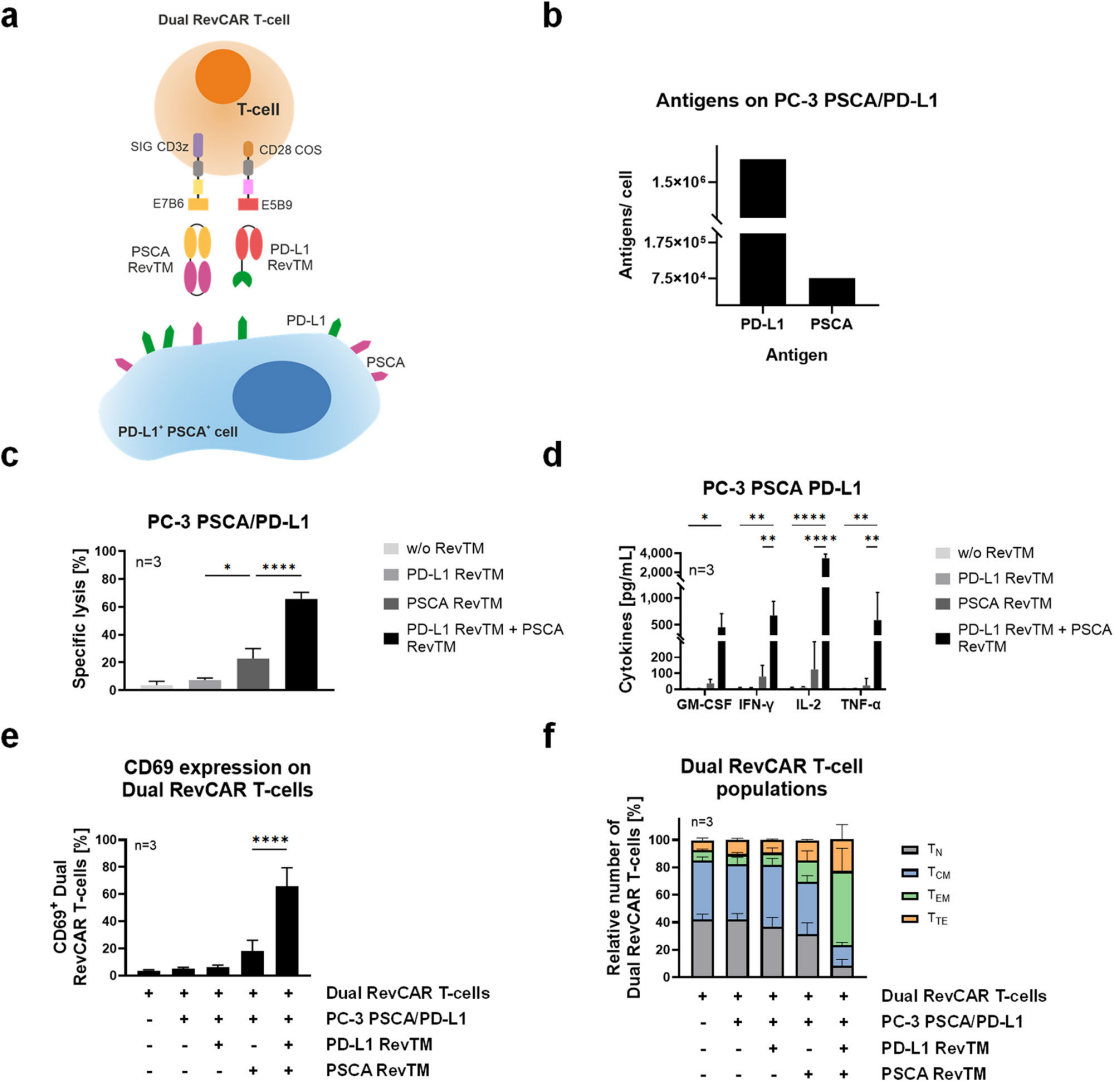
AND-gated targeting of PC-3 cells expressing both PSCA and PD-L1 using Dual RevCAR T-cells and two different target modules. **a** Dual RevCAR T-cells express two separate receptors: the signaling (SIG) RevCAR-E7B6 triggering the CD3z signal and the costimulatory (COS) RevCAR-E5B9 triggering the CD28 co-stimulatory signal. The SIG contains intracellular CD3z signaling domain (purple), transmembrane domain (gray), hinge domain (yellow), and peptide epitope E7B6 (dark yellow). The COS comprises intracellular CD28 domain (brown), transmembrane domain (gray), hinge domain (pink), and peptide epitope E5B9 (red). Dual RevCAR T-cells are redirected to PSCA^+^ PD-L1^+^ cancer cells via PSCA RevTM and PD-L1 RevTM. The simultaneous recognition of PSCA and PD-L1 by SIG and COS RevCARs via the respective RevTMs triggers both CD3z and CD28 signals resulting in complete activation of Dual RevCAR T-cells. **b** Comparative expression of PD-L1 and PSCA on the surface of PC-3 PSCA/PD-L1. **c**, **d** 5 × 10^3^ PC-3 PSCA/PD-L1 cells were co-cultured for 24 h with Dual RevCAR T-cells (E:T = 5:1) in the absence of RevTM, in the presence of only PD-L1 or PSCA RevTM, or in the presence of both RevTMs. **c** Specific lysis and **d** cytokine secretion were determined. **e**, **f** Dual RevCAR T-cells were seeded in the absence of target cells, in the presence of target cells alone, in the presence of target cells and either PD-L1 RevTM or PSCA RevTM, or in the presence of target cells and both RevTMs, for 24 h (E:T = 5:1). Then, the T-cells were stained with **e** anti-CD69 antibody or **f** anti-CD62L and anti-CD45RO antibodies. Dual RevCAR T-cells were divided into four groups of increasing differentiation: Naive (T_N_): CD62L^high^ and CD45RO^low^; Central memory (T_CM_): high expression of both markers; Effector memory (T_EM_): CD62L^low^ and CD45RO^high^; and Terminal effector (T_TE_): low expression of both markers. **c**–**f** Data for three individual T-cell donors are shown as mean ± SD. **c**–**e**, statistical significance was assessed by ordinary one-way ANOVA (**c**, **e**) or two-way ANOVA (**d**) followed by Tukey’s multiple comparisons test; *P* ≤ 0.05 (*), *P* ≤ 0.01 (**), *P* ≤ 0.0001 (****).

To achieve higher specificity and reduce the risk for on-target-off-tumor effects, we aimed to apply an AND-gated targeted strategy by combining the PD-L1 RevTM and another tumor-specific RevTM. For proof of concept, we chose the PC-3 PSCA/PD-L1 cell model and the TAA PSCA as an additional target. In contrast to second-generation RevCAR T-cells, in the Dual RevCAR T-cells the signaling and costimulatory domains are divided into two RevCAR constructs, each containing a different epitope of the nuclear La/SS-B protein (Fig. 7a). The signaling CAR (SIG) contains the activation domains of CD3 zeta chain in the intracellular domain and the E7B6 epitope in the extracellular domain. The co-stimulatory CAR (COS) contains the CD28 signaling domains intracellularly and the E5B9 epitope extracellularly. The complete activation of Dual RevCAR T-cells is achieved by the simultaneous binding of both RevTMs to their respective antigens and to both signaling and costimulatory RevCARs of the Dual RevCAR T-cells, triggering both CD3 and CD28 signals (Fig. 7a). The PD-L1 RevTM contains an scFv with specificity for the E5B9 epitope, and therefore is able to provide the co-stimulatory signal. To activate the signaling RevCAR, an already-established anti-PSCA RevTM binding to PSCA and the E7B6 epitope^14^ was selected (Fig. 7a). The expression of PD-L1 and PSCA on PC-3 PSCA/PD-L1 was quantified, showing that 1.8 × 10^6^ molecules of PD-L1 can be found on the surface of these cells, compared to 7.5 × 10^4^ of PSCA (Fig. 7b). Next, to assess the functionality of the dual AND-gated targeting strategy, PC-3 PSCA/PD-L1 were co-cultured with Dual RevCAR T-cells and either no RevTM, only one, or both PD-L1 and PSCA RevTMs (Fig. 7c). No significant lysis was observed in the conditions without RevTM or with only PD-L1 RevTM, and only marginal lysis in the presence of PSCA RevTM alone. However, when both RevTMs were present, we could see an effective and highly significant killing. To characterize the redirected Dual RevCAR T-cells, we assessed the secretion of pro-inflammatory cytokines (Fig. 7d), CD69 expression (Fig. 7e), and memory phenotypes (Fig. 7f). We observed that a significant cytokine secretion only takes place in the presence of both RevTMs. The same is true for the expression of the early activation marker CD69. Regarding the phenotype of the Dual RevCAR T-cells, a considerable shift towards effector phenotypes is only achieved when both RevTMs are present. As a whole, we can see a complete activation of the RevCAR T-cells upon the combinatorial targeting of PSCA and PD-L1, not only in terms of T-cell activation (CD69 expression) and tumor cell killing but also in the secretion of pro-inflammatory cytokines and T-cell differentiation, portraying an AND-gated tumor targeting using the RevCAR system.

### Targeting of PD-L1 via the RevCAR and Dual RevCAR T-cell system in vivo

**Fig. 8:**
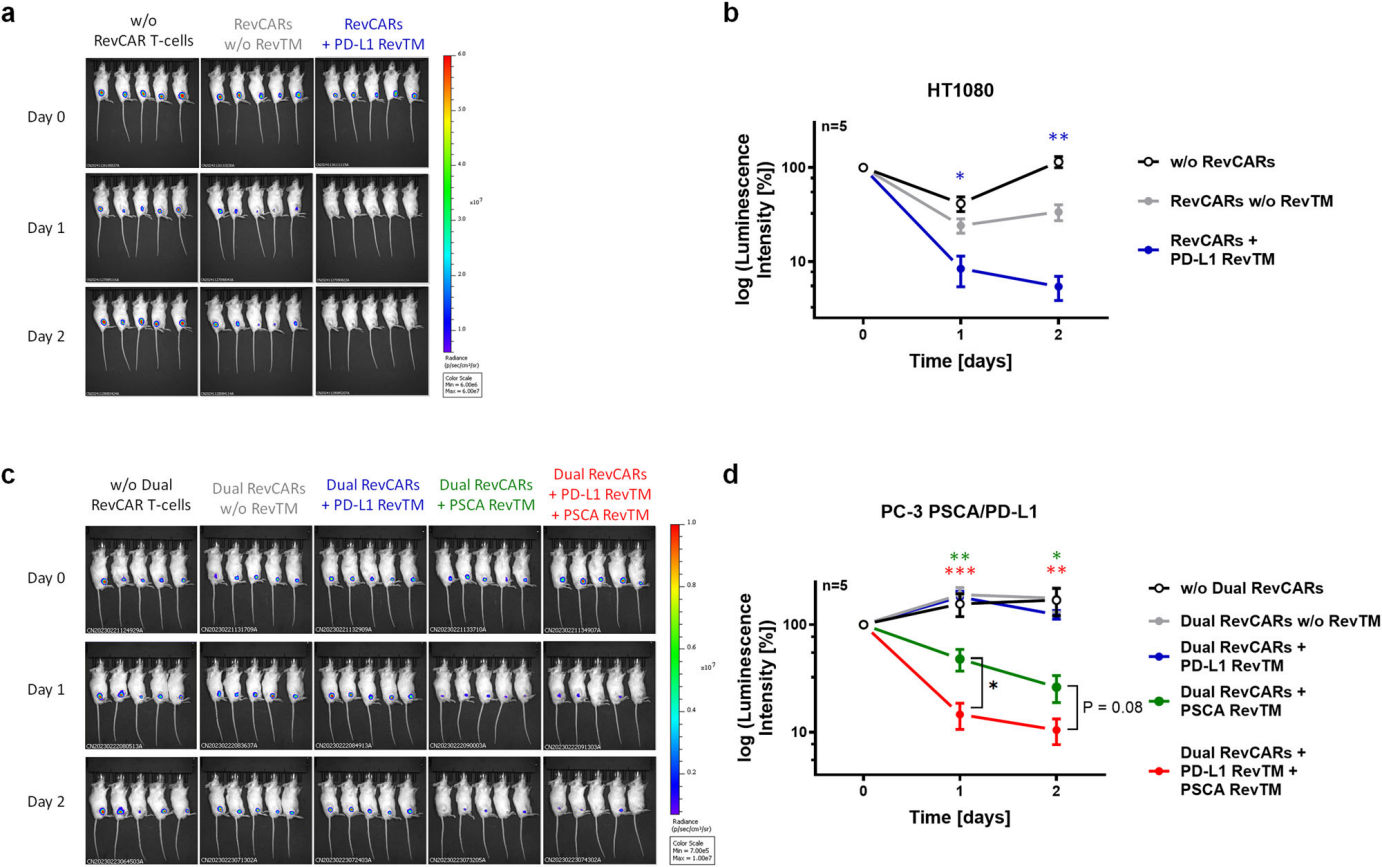
In vivo functionality of the RevCAR and Dual RevCAR T-cell system targeting PD-L1 singly or both PD-L1 and PSCA on tumor cells, respectively. **a** Female NXG mice were s.c. injected with HT1080 tumor cells either in the absence of RevCAR T-cells (black), in the presence of RevCAR T-cells only (gray), or in the presence of both RevCAR T-cells and PD-L1 RevTM (blue). Bioluminescence signals at day 0 (day of injection), day 1 and day 2 are shown. **b** The intensity of bioluminescence was quantified and represented as mean ± SD for five individual mice. Statistical analysis was performed compared to the control group (RevCARs w/o RevTM, gray) using Student’s *t* test; *P* ≤ 0.05 (*), *P* ≤ 0.01 (**). **c** Male NXG mice were s.c. injected with PC-3 PSCA/PD-L1 tumor cells either alone (black), in the presence of Dual RevCAR T-cells (gray), or in the presence of Dual RevCAR T-cells and the following RevTMs: PD-L1 RevTM (blue), PSCA RevTM (green) or both PD-L1 RevTM and PSCA RevTM (red). Bioluminescence signals at day 0, day 1 and day 2 are shown. **d** The intensity of bioluminescence was quantified and represented as mean ± SD for five individual mice. Statistical analysis was performed compared to the control group (Dual RevCARs without RevTM, gray) or comparing specific groups as shown using Student’s *t* test; *P* ≤ 0.05 (*), *P* ≤ 0.01 (**), *P* ≤ 0.001 (***).

After a successful in vitro validation, we tested our novel PD-L1 targeting RevCAR or Dual RevCAR T-cell approaches in vivo using a xenograft immunodeficient mouse model. For proof of concept of the killing of PD-L1-expressing tumor cells by the novel anti-PD-L1 RevCAR T-cell system in vivo, we conducted the experiment with three different groups of five NXG mice each. As target cells, we used HT1080 since it is a well-established model that natively expresses PD-L1 and can engraft successfully in mice. In the first group, we injected HT1080 tumor cells alone subcutaneously into the flank; in the second group, tumor cells and RevCAR T-cells; and in the third group, tumor cells, RevCAR T-cells and PD-L1 RevTM. Tumor growth was monitored by bioluminescence imaging (Fig. 8a, b). After one day, the tumor growth was significantly decreased in mice that received RevCAR T-cells with PD-L1 RevTM compared to the group that received only RevCAR T-cells in the absence of PD-L1 RevTM. After two days, this effect was even more pronounced.

To test whether the Dual RevCAR T-cell approach kills PD-L1- and PSCA-double positive tumor cells in vivo upon recognition of both antigens at the same time, an additional mouse experiment was conducted, consisting of five different groups with five mice each. Since our HT1080 model lacks PSCA expression, we used PC-3 PSCA/PD-L1 cells instead that recombinantly express both PSCA and PD-L1. Mice were subcutaneously injected with PC-3 PSCA/PD-L1 tumor cells, either alone or in the presence of Dual RevCAR T-cells. In the latter, either no RevTM, PD-L1 RevTM, PSCA RevTM, or both RevTMs were added (Fig. 8c, d). One day after co-injection, the group where Dual RevCAR T-cells and both RevTMs were present displayed a significant reduction in tumor size compared to the control group with only Dual RevCAR T-cells and tumor cells. The group with PSCA RevTM alone also showed a significant decrease in the tumor signal in comparison to the control group with tumor and RevCAR T-cells, most likely due to the slight activation of the first-generation SIG RevCAR via the PSCA RevTM upon PSCA recognition, which was also observed in vitro but to a lesser degree (Fig. 7c). However, the group with both RevTMs showed a significantly stronger reduction in tumor growth than the group with PSCA RevTM alone. Two days after injection, the tumor size for these two groups decreased further.

## Discussion

CAR T-cell therapy is an attractive strategy for cancer treatment, but it still needs to be optimized for solid tumors^9,21^. The modular RevCAR system can increase safety and flexibility, but the challenges of the immunosuppressive TME still persist^14^. A promising strategy would be to target immune checkpoints such as PD-L1, often upregulated by tumor cells and their microenvironment^24^. To target PD-L1-expressing cells, we designed a protein-based bispecific RevTM for an application with the RevCAR system, termed PD-L1 RevTM, and performed a deep characterization of its functionality in combination with the immunotherapeutic RevCAR platform. A flow-cytometry-based binding assessment showed specific binding of PD-L1 RevTM to PD-L1-expressing cell lines, in particular to the PC-3 PSCA/PD-L1 cells, which were modified to overexpress PD-L1. For four naturally PD-L1-expressing cell lines, MDA-MB-231, U87MG, HT1080, and SCP-1, we could not detect any binding. A quantitative analysis of PD-L1 expression revealed that the artificially expressing PC-3 cells have 300- to 1200-fold more antigens on their surface compared to the other cell lines. This may explain why the binding was detected for this cell line only, where the affinity of the PD-L1 RevTM was calculated in the low nM range. Regardless, we showed a significant and specific killing of all five PD-L1-expressing cell lines only in the presence of both RevCAR T-cells and PD-L1 RevTM. We can, therefore, conclude that the necessary binding is taking place under the conditions used in such cytotoxicity assays, even if not measurable by flow cytometry. This is in agreement with previously published data, where a significant killing was observed despite the undetectable binding^33,34^. To investigate the potency of the system, we tested its performance under various E:T ratios. For the four cell lines tested, we found out that efficacy was not compromised at an E:T ratio up to 1:2. Overall, the anti-tumor functionality, even at low E:T ratios, highlights the efficiency and sensitivity of our PD-L1-targeting RevCAR T-cells system. In this regard, also the novel PD-L1 RevTM works highly efficiently in the pM to nM range, showing a promising therapeutic potential. Although EC_50_ values remained relatively constant at different time points, the maximum killing increased at 48 h compared to 24 h, which might be explained simply by prolonged incubation time and/or an upregulation of PD-L1 taking place over time upon the co-culture, here exemplarily, demonstrated for SCP-1 cells by multiplex immunohistochemistry staining. This finding is in accordance with other studies showing that PD-L1 expression is often upregulated in the presence of IFN-γ^35^, which we showed to be secreted by the RevCAR T-cells in the co-culture setting. The secretion of pro-inflammatory cytokines, expression of CD69, and the shift towards an effector T-cell phenotype confirm that the RevCAR T-cells become specifically activated and cytotoxic upon co-culture with PD-L1-expressing target cells in the presence of PD-L1 RevTM. These findings were proven and confirmed using three different target cell lines expressing different PD-L1 levels which might correlate with the intensiveness of the immune responses. Although killing efficacy was comparably high across three different target cell lines regardless of their entity and PD-L1 expression density, the concentration of cytokines secreted and degree of activation marker CD69 expression varied in a more pronounced way. Consequently, we concluded that various RevCAR T-cell functions depend on multiple different factors. As previously reported by others, the effector to tumor cell interaction can vary due to various factors such as the tumor cell entity, antigen density, and the presence of immunomodulatory surface molecules, influencing the T-cell functions^36^. Overall, RevCAR T-cells show a robust response across different entities and expression levels of PD-L1, which can vary widely in solid tumors.

Besides confirming an effective PD-L1-dependent killing and T-cell activation in monolayer cells by PD-L1 RevTM redirected RevCAR T-cells, we also observed functionality against 3D tumor models. Our data showed that SCP-1 spheroids could be successfully killed by redirected RevCAR T-cells, whose activation led to a controlled and specific release of pro-inflammatory cytokines. In addition, immunofluorescence analysis revealed a specific RevCAR T-cell infiltration into the spheroids and upregulation of several activation markers in the presence of RevTM. We also detected that in the presence of RevCAR T-cells and PD-L1 RevTM, SCP-1 cells in the spheroid upregulate PD-L1 expression. In addition to these spheroids derived from a cell line, we also tested the approach in patient-derived tumor spheroids from liposarcoma, which more closely relate to and often retain some of the features of the original tumors^32^. Using such models, we could confirm a specific lysis of PD-L1-expressing spheroids accompanied by pro-inflammatory cytokine secretion, underlining the clinically relevant immunotherapeutic potential of the novel PD-L1-directed RevCAR T-cell approach.

In addition to the in vitro experiments, a proof of concept for the functionality of the PD-L1 targeting RevCAR T-cell approach was also demonstrated in vivo. Significant tumor growth suppression was observed in the group where both the RevCAR T-cells and PD-L1 RevTM were present, compared to the control group with the RevCAR T-cells alone. A slight, non-significant tumor signal reduction was also seen in mice injected with RevCAR T-cells in the absence of RevTM, likely due to alloreactions of the human RevCAR T-cells against the xenotransplanted tumor cells in mice independent from the RevCARs but rather mediated via the TCR. As already observed in vitro, this alloreaction highly varies between different T-cell donors. Another limitation of this allogeneic mouse xenograft model^37^ is that it allows only a short-term anti-tumor study due to the short half-life of small RevTMs such as the PD-L1 RevTM, which are rapidly eliminated through the renal system within minutes^19^. To explore long-term anti-tumor effects in vivo, a continuous or subsequent injection of the PD-L1 RevTM would be required which will be the focus of prospective in vivo studies. However, our results demonstrate that the RevCAR T-cells redirected by the PD-L1 RevTM can efficiently kill PD-L1-expressing tumor cells in vivo.

Taking advantage of the versatility of the RevCAR system, higher specificity may be achieved using a logic-gated targeting approach, employing Dual RevCAR T-cells. In this context, we used an AND-gated targeting of PD-L1 and the TAA PSCA on PC3-PSCA/PD-L1 cells to increase the specificity for on-target-on-tumor targeting and reduce the risk of on-target-off-tumor effects. Dual RevCAR T-cells have the CD3z and CD28 signaling split into two separate RevCAR constructs, requiring the simultaneous binding of two different RevTMs to the target cells for full activation. It must be considered that the signaling RevCAR containing only the canonical CD3z signaling is equivalent to a first-generation CAR which is still functional on its own, albeit to a small degree. On the other hand, the RevCAR, which provides CD28 co-stimulation alone in the absence of CD3z activation, is not sufficient to trigger a T-cell response. Therefore, we chose to use PD-L1 for the co-stimulatory signal, so that in the absence of the TAA of interest, there would be no killing of PD-L1-expressing cells. For CD3z signaling, we targeted PSCA, an established TAA, with a previously developed RevTM^14^. In this case, even though the target cells had an extremely high PD-L1 expression, the presence of the PD-L1 RevTM alone didn’t lead to any lysis or activation of the Dual RevCAR T-cells. The presence of PSCA RevTM, on the other hand, marginally activated the CD3z signaling triggered via the first-generation SIG RevCAR of the Dual RevCAR T-cells, leading only to minimal killing in vitro as well as in vivo but not resulting in a significant activation or release of pro-inflammatory cytokines in vitro. Both in vitro and in vivo, the effect of the AND-gated tumor targeting could be achieved only in the presence of both RevTMs recognizing both tumor antigens, resulting in significantly higher specific lysis and overall activation of the Dual RevCAR T-cells. It is worth highlighting that the system is functional despite using two RevTMs with different formats: in the PSCA RevTM two scFvs are connected in a tandem format, while the PD-L1 RevTM contains an scFv linked to a fragment of the PD-1 protein. As a matter of fact, we have already shown that dual targeting is possible even when different formats and sizes are used, such as the combination of nanobody with scFv or IgG4 formats and IgG4 with minibody formats^19,20^.

Ultimately, we have developed a novel strategy for targeting the immune checkpoint PD-L1 in solid tumors. In contrast to immune checkpoint blockade^25,26^ or other alternatives that aim only to overcome PD-L1 immunosuppression^28–30^, this approach targets PD-L1-expessing cells directly. Compared to conventional PD-L1 CARs^31^, this system is switchable, turning an immunosuppressive marker into a T-cell-activating one in a safe and reversible way. Employing PD-L1 as the target moiety for RevCAR T-cells might be beneficial by additionally providing a positive feedback loop that could result in increased tumor cell killing over time, such as has already been described in other PD-L1 CARs^31^. The RevCAR system would allow a rapid intervention and control of the RevCAR T-cells and their respective killing activity due to the short half-life of small RevTMs^19^ like the PD-L1 RevTM. We have demonstrated the functionality of the PD-L1 RevTM using the RevCAR system across different models of increasing complexity and diverse solid tumor entities, in vitro as well as in vivo. We have also shown that a combinatorial AND-gated tumor targeting approach of a TAA and PD-L1 is possible, and may be beneficial depending on the characteristics of the tumor and the patient. Most likely at a later treatment phase, when the tumor burden is low, the AND-gated Dual RevCAR T-cell approach could enable a precise attack of only the tumor cells while saving the healthy ones. Previously, we established and preclinically proved an AND-gated functionality of the Dual RevCAR T-cell approach targeting several different antigen pairs and various tumor entities (including hematological and solid tumors) in vitro and in vivo, using cell lines as well as patient-derived materials^14,18–20^. This confirms the reliability and robustness of our Dual RevCAR targeting system, despite its complexity and dependence on many different factors, including antigen density, RevCAR expression level, RevCAR signal strength, and tumor entity^14,18–20^. This study supports the notion that the RevCAR system, in its variations, could be a suitable therapeutic platform for the modulation of the TME and a precise PD-L1 targeting of solid tumors.

## Methods

### Cell culture

The cell lines HT1080, HEK-293T, and 3T3, as well as the prostate cancer cell line PC-3 and glioblastoma cell line U87MG, were obtained from the American Type Culture Collection. The human breast cancer cell line MDA-MB-231 was obtained as previously described^38^, and the immortalized mesenchymal cell line SCP-1 was gently provided by Dr. Martin Bornhäuser and Dr. Manja Wobus (Department of Clinical Medicine 1, University Hospital Carl Gustav Carus, TU Dresden). PC-3 cells were genetically modified by lentiviral transduction to express PSCA as described before^39^ and with the open reading frame (orf) encoding human PD-L1, resulting in a new cell line we named PC-3 PSCA/PD-L1. Cell lines PC-3 PSCA/PD-L1, MDA-MB-231, HT1080, U87MG, and SCP-1 were subsequently transduced with the gene encoding firefly luciferase (Luc) for luciferase-based cytotoxicity assays. PC-3, PC-3 PSCA, and PC-3 PSCA/PD-L1 cells were cultured in RPMI 1640 medium completed with 10% FCS, 100 U/mL penicillin, and 100 µg/mL streptomycin, 2 mM N-acetyl-L-alanyl-L-glutamine, 1% non-essential amino acids and 1 mM sodium pyruvate (Biochrom). The rest of the cell lines were cultured in DMEM supplemented with 10% FCS, 100 µg/mL penicillin/streptomycin, and 1% nonessential amino acids (Sigma Aldrich). All cells were kept at 37 °C in a humidified atmosphere of 5% CO_2_. SCP-1 spheroid formation was performed when necessary, as previously described^40^. Spheroid images were taken with the Keyence microscope BZ-X710.

### Patient-derived spheroids

Two patient-derived sarcoma spheroid cultures, HLS1 and HLS1, were generated (patient information can be found in Supplementary Table 2). Sarcoma tissue was minced into small pieces (<2 mm) and enzymatically dissociated with 2 mg/mL collagenase IV in Medium 199 (Invitrogen) containing 150 µM CaCl2 for 2.5 h. Subsequently, purified tumor cells were washed with D-PBS (Invitrogen) and successively filtered through 100 µm and 40 µm cell strainers (BD Biosciences) to obtain a single cell suspension. To establish spheroids cultures, singularized cells were seeded into ultra-low attachment plates (Corning) in Advanced DMEM/F12 medium supplied with 3 g glucose, 2 mM L-glutamine, 1% penicillin/streptomycin, B27 supplement (Invitrogen), 5 mM HEPES buffer, 6 mg heparin (Sigma- Aldrich), 10 ng/mL FGF2, 20 ng/mL FGF10, and 20 ng/mL Nodal (R&D Systems). Established spheroids were cultured into serum-free medium in ultra-low attachment plates (Corning) in the same medium of culture establishment. The medium was changed at least once a week, and cytokines were supplemented three times per week. When the medium turned yellow or more than 80% of the spheroids showed a necrotic center, they were split at 1:3 to 1:6 ratio. Enzymatic dissociation of spheroids to cell clumps was done using 1 mL Trypsin 0,25% EDTA (Life Technologies) for 15 to 60 min at 37 °C, avoiding mechanical stress. To stop the dissociation, 2 mL PBS 20% FCS (PAN-Biotech) was added, and the cells were centrifuged and washed with PBS before re-seeding. Sarcoma spheroids were regularly tested for bacteria and virus contaminations with the Mutiplex Cell Contamination test, and for intra-human cell contaminations via Human Cell Line Identification (Heidelberg, Germany)^41^. For luciferase-based cytotoxicity assays, sarcoma spheroids were lentivirally transduced as described before^39^ with the gene encoding firefly luciferase (Luc). Studies with patient-derived sarcoma samples were approved by the Ethics Committee of the Medical Faculty of Heidelberg University (S-206/2011) and by the Medical Faculty Carl Gustav Carus, Technical University Dresden, Germany (EK 431102015). All studies using human participants or samples have complied with all relevant ethical regulations including the Declaration of Helsinki.

### Production of RevCAR T-cells

Human PBMCs were isolated by Pancoll gradient centrifugation using LeucoSEP^TM^ tubes from buffy coats of healthy blood donors who provided written consent (German Red Cross, Dresden, Germany). T-cells were then obtained by magnetic separation with the Miltenyi Biotec Pan T-cell Isolation Kit following the instructions of the manufacturer. RevCAR-E5B9-28/3z T-cells (in this manuscript referred to as RevCAR T-cells) or Dual RevCAR T-cells were generated by lentiviral transduction, expanded, and cultured as previously described^14^. Transduction efficiency was assessed by the reporter gene GFP and by the binding of anti-La (5B9) antibody. The studies with human T-cells were approved by the Medical Faculty Carl Gustav Carus, Technical University Dresden, Germany (EK138042014). All studies using human participants or samples have complied with all relevant ethical regulations including the Declaration of Helsinki.

### Design, expression, and purification of PD-L1 RevTM

In order to target PD-L1-expressing cells, a novel RevTM was designed. It contains the extracellular region of the PD-1 protein, which naturally binds to PD-L1, and the anti-La scFv (5B9) that binds to the E5B9 peptide epitope on the RevCARs. For simple purification, an SP for extracellular secretion and a 6xHis tag were also included. 3T3 cells were transduced with the lentiviral vector encoding the RevTM of interest and the cell culture supernatant was collected, followed by Ni-NTA affinity chromatography (QIAGEN). The purified RevTM was subsequently analyzed by SDS-PAGE and western blot.

### Flow cytometry-based binding assays

The binding capacity of the PD-L1 RevTM to target cells (PC-3 PSCA, PC-3 PSCA/PD-L1, MDA-MB-231, and SCP-1) and T-cells was determined via flow cytometry. For this, 2 × 10^5^ cells were incubated with the RevTM for 1 h at 4 °C, washed, and then incubated again for 30 min at 4 °C with anti-His-PE mAb (Miltenyi Biotec GmbH). The stained cells were measured with the MacsQuant10 Analyzer (Miltenyi Biotec GmbH), and the results were analyzed with MACSQuantify Software (Miltenyi Biotec GmbH). The same strategy was used to determine PD-L1, EGFR, or RevCAR expression, using the purified anti-PD-L1 purified mAb (Biolegend), purified anti-EGFR mAb (Biolegend), or purified anti-La(5B9) mAb (Davids) as a primary antibody and goat anti-mouse Pacific Blue™ mAb (Invitrogen) as the secondary. The purified mouse IgG2b, κ isotype control antibody (Biolegend) was used as a negative control. To determine antigen density on the cells’ surface, the Quantitative Analysis Kit QIFIKIT® was used (Agilent) following the manufacturer’s instructions.

### Cytotoxicity assays

The cytotoxicity induced by the RevCAR T-cells was analyzed by chromium-release assay^39^ or luciferase-based assay^42^, both of which were previously described. Briefly, target cells that were either pre-incubated with ^51^Cr or previously transduced with luciferase were seeded into a 96-well plate. In the case of spheroids, spheroid formation was performed for 24 or 48 h prior to the killing assay. Different conditions were performed in triplets, such as the presence or absence of RevCAR T-cells and the presence or absence of RevTM, and the readout was done after 24 h or 48 h. The concentration of RevTM used for most experiments was 50 nM, except for EC_50_ value calculation, where different concentrations of PD-L1 RevTM were used. An effector-to-target (E:T) ratio of 5:1 was used, unless stated otherwise, with 5 × 10^3^ target cells and 2.5 × 10^4^ T-cells per well.

### Cytokine release assays

To determine the concentration of cytokines released into co-culture supernatants, the MACSPlex Cytokine 12 Kit (Miltenyi Biotec GmbH) was used following the manufacturer’s instructions. The measurement was done with a MACSQuant Analyzer 10 (Miltenyi Biotec GmbH) and the data were analyzed with the MACSQuantify^®^ software (Miltenyi Biotec GmbH).

### T-cell activation and memory phenotyping

The activation and memory status of RevCAR T-cells was analyzed by doing a 24 h co-culture of RevCAR T-cells with or without target cells and with or without 50 nM RevTM at an E:T ratio of 5:1. After the co-culture, the T-cells of each condition were collected and incubated for 20 min at 4 °C with either anti-CD69-APC mAb (Miltenyi Biotec GmbH) in the case of activation staining or anti-CD45RO-PE mAb (Miltenyi Biotec GmbH) and anti-CD62L-Pacific Blue™ mAb (Biolegend) in the case of memory staining. For the analysis, we gated on EGFP^+^ cells, since this is a reporter gene for the RevCAR and Dual RevCAR constructs.

### Spheroid multiplex immunohistochemistry staining

SCP-1 spheroids were seeded at a density of 6 × 10^4^ cells per spheroid and co-cultured with RevCAR T-cells at an E:T ratio of 5:1 in the presence or absence of 50 nM RevTM. The spheroids were fixed with 4% neutral buffered formalin (NBF) for 2 h, counterstained with hematoxylin, and embedded in histogel (Fisher Scientific). Subsequently, they were fixed overnight in 4% NBF before dehydration and paraffin embedding. Using a microtome, the spheroids were cut into 2.5 µm sections and mounted onto microscope slides (Agilent Dako). Multiplex immunohistochemistry (mIHC) was performed using the Opal System (Akoya Biosciences) on the Ventana Discovery Ultra platform (Ventana Medical Systems) as described in detail, previously^43^. In short, the initial steps involved deparaffinization and heat-mediated antigen retrieval, followed by incubation with the primary antibody. A suitable horseradish peroxidase (HRP)-coupled OmniMap secondary antibody (Ventana Medical Systems) was then applied, followed by incubation with an Opal TSA fluorophore (Akoya Biosciences). The HRP converts the Opal fluorophore, which covalently binds to tyramide residues, resulting in light emission within the fluorophore’s spectrum. This signal remains stable through the subsequent heat-mediated stripping conducted after each staining cycle to remove antibody complexes. Up to six different markers were detected by repeating the protocol from the incubation of the primary antibody until the heat-mediated stripping of the antibody complexes. The individual dilutions, fluorophore couples, and the suppliers of each antibody are listed in Supplementary Table 1. Subsequent to the final staining cycle, the slides were counterstained with DAPI (Sigma-Aldrich) and mounted in Fluoromount-G® medium (SouthernBiotech). Whole-slide scans of the sections were captured using the Vectra 3.0 Automated Imaging System (Akoya Biosciences), and regions of interest were identified utilizing Phenochart™ software (Akoya Biosciences). Multispectral images (MSIs) at ×200 magnification were acquired, spectrally unmixed, and exported as multi-channel TIFF files using inForm software (Akoya Biosciences). These images were imported into QuPath software^44^ in which a pixel classifier, cell segmentation using the Stardist QuPath extension^45^, and a composite object classifier were trained to phenotype all detected cells. Data obtained from QuPath were analyzed using packages from the tidyverse collection in RStudio and R v.2.2.2^46^ to evaluate the infiltration and activation status of the RevCAR T-cells. The image processing for representative images of the staining occurred using ImageJ software^47^.

### Tumor xenograft models

For in vivo experiments, the experimental protocols, including general animal husbandry were approved by the local Ethical Committee for Animal Experiments (AZ 25-5131/562/49 and DD24.1-5131/449/67, Landesdirektion Sachsen, Chemnitz, Germany). The experiments were carried out at the animal facility at Helmholtz-Zentrum Dresden-Rossendorf (HZDR, Dresden, Germany) in accordance with relevant guidelines and regulations, earliest one week after transport to ensure acclimatization. Animals were kept under specific pathogen-free conditions following FELASA recommendations in type-II L cages individually ventilated (type-II L) adhering to the requirements established by European Directive 62/2010 regarding cage dimensions with five mice per cage. All rooms were kept at 22–26 °C, 40–70% humidity, and 12/12 hours of day/night lighting, including the dawn phase. The animals were provided autoclaved bedding, certified irradiated food, acidified drinking water ad libitum, nesting material, and rodent tubes as enrichments. The hygiene status of the animals was guaranteed by specific-pathogen-free animal housing, separate area clothing, and additional protective clothing (hood, face mask, gloves, lab coat, galoshes, or separate area shoes). Monitoring by sentinel animals (immune-competent mice from breeder companies) as well as semi-annual hygiene inspection according to FELASA-guidelines are implemented.

To assess the anti-tumoral effect of RevCAR T-cells in combination with PD-L1 RevTM, 8-week-old female NXG mice (Janvier Labs) were subcutaneously (s.c.) injected in the right flank with 5 × 10^5^ luciferase-expressing HT1080 cells either alone, in the presence of RevCAR T-cells (E:T = 1:1), or in the presence of both RevCAR T-cells and 300 pmol PD-L1 RevTM. Each group consisted of five mice and the mixture was injected in a final volume of 100 µL of PBS per mouse. For the Dual RevCAR system, the experiment was performed with 9-week-old male NXG mice (Janvier Labs). Experimental groups consisted of five mice which were s.c. injected in the right flank with 1 × 10^6^ PC-3 PSCA/PD-L1 cells either alone or in combination with Dual RevCAR T-cells at a ratio of 1:1. Depending on the group, the mixture also included either no RevTM, PD-L1 RevTM, PSCA RevTM or both PD-L1 RevTM and PSCA RevTM (each 300 pmol per mouse). To improve tumor cell engraftment, the final mixture was injected in a 1:1 solution of PBS and Matrigel (Corning), at a final volume of 100 µL of PBS per mouse. For both experiments, anti-tumor effects were measured by detecting luciferase-positive tumor cells by optical imaging of luminescence signals in relative luminescence units (RLU). For this, mice were anesthetized (7–9% Desfluran, 300 ml/min O_2_, 700 ml/min Air) and s.c. injected in the neck fold with 150 µL of luciferin solution (Luciferin Potassium salt, #122799, Revvity Germany Diagnostics GmbH, working solution: 15 mg/ml in PBS). Following an incubation time of 10 minutes, bioluminescence was measured by means of an IVIS Spectrum In Vivo Imaging System (PerkinElmer). Data analysis was performed by using the Living Image Software (PerkinElmer).

All animals were monitored (general condition, weight, movement or behavioral abnormalities, tumor size) by qualified personnel according to the approved score sheets. After the experiment was completed, the animals were euthanized under anesthesia by cervical dislocation. For sample size calculation, the minimum number of animals per group was chosen to ensure that the results were meaningful and achieved statistical significance between experimental groups. At least five mice per group (treatment and control groups) are needed to reliably calculate the average of RLU, also confirmed by previous data^19,20^. For both experiments, sex, and age-matched mice were randomly allocated to the experimental group and marked by ear holes. In each cage, only mice of the same experimental group were kept. Researchers were aware of the group allocation throughout the experiment and data analysis and there were no animal or data point exclusions.

### Statistical analysis

Statistical analysis was performed using GraphPad Prism 10.1.2 (GraphPad Software), and statistical significance was determined as indicated in the figure legends. *P* values equal to or lower than 0.05 were considered significant as follows: *P* ≤ 0.05 (*), *P* ≤ 0.01 (**), *P* ≤ 0.001 (***), *P* ≤ 0.0001 (****).

## Supplementary information

RevCAR-mediated T-Cell Response against PD L1-expressing Cells Turns Suppression into Activation-SUPPLEMENTAL MATERIAL

## Data Availability

The datasets used and/or analyzed during the current study are available from the corresponding author upon reasonable request.
