# Supplementary material for: RevCAR-mediated T-cell response against PD-L1-expressing cells turns suppression into activation
Source: NPJ Precis Oncol. 2025 Feb 9;9:42. doi: 10.1038/s41698-025-00828-6 (PMC11808103; doi:10.1038/s41698-025-00828-6)
Supplement: Supplementary file 1 — RevCAR-mediated T-Cell Response against PD L1-expressing Cells Turns Suppression into Activation-SUPPLEMENTAL MATERIAL [file 41698_2025_828_MOESM1_ESM.pdf]

RevCAR-mediated T-Cell Response against PD-L1-expressing Cells Turns Suppression into Activation

Supplementary data

Supplementary Table 1. Antibodies and fluorophores used for multiplex immunohistochemistry staining of spheroids.

Antibody	Source	Clone	Dilution	Fluorophore	Dilution
CD3	Roche	2GV6	prediluted	Opal 520	1:75
GrzB	Agilient Dako	Grb-7	1:200	Opal 570	1:400
Ki67	Agilient Dako	MIB-1	1:75	Opal 690	1:75
PD-1	Roche	NAT105	prediluted	Opal 620	1:250
LAG-3	Cell Signaling Technologies	D2G4O	1:125	Opal 540	1:100
PD-L1	Cell Signaling Technologies	E1L3N	1:75	Opal 650	1:75

Supplementary Table 2. Overview of patient information used to generate sarcoma *in vitro* 3D cultures.

Culture name	Origin in patient	Sarcoma subtype	Age	Sex	Stage
HLS1	Retroperitoneum	Dedifferentiated liposarcoma	73	Male	IIIa
HLS2	Retroperitoneum	Liposarcoma	39	Male	IIIa
